# The Effects of Host Plant Genotype and Environmental Conditions on Fungal Community Composition and Phosphorus Solubilization in Willow Short Rotation Coppice

**DOI:** 10.3389/fpls.2021.647709

**Published:** 2021-07-05

**Authors:** Piotr Koczorski, Bliss Ursula Furtado, Marcin Gołębiewski, Piotr Hulisz, Christel Baum, Martin Weih, Katarzyna Hrynkiewicz

**Affiliations:** ^1^Department of Microbiology, Faculty of Biological and Veterinary Sciences, Nicolaus Copernicus University, Torun, Poland; ^2^Department of Plant Physiology and Biotechnology, Faculty of Biological and Veterinary Sciences, Nicolaus Copernicus University, Torun, Poland; ^3^Interdisciplinary Center for Modern Technologies, Nicolaus Copernicus University, Torun, Poland; ^4^Department of Soil Science and Landscape Management, Faculty of Earth Sciences and Spatial Management, Nicolaus Copernicus University, Torun, Poland; ^5^Soil Science, Faculty of Agricultural and Environmental Sciences, University of Rostock, Rostock, Germany; ^6^Department of Crop Production Ecology, Swedish University of Agricultural Sciences, Uppsala, Sweden

**Keywords:** diversity, fungal endophytes, phosphate solubilization, rhizosphere fungi, short rotation cropping, willow

## Abstract

Phosphorus (P) is an essential plant nutrient. Low availability of P in soil is mainly caused by high content of Fe_2_O_3_ in the clay fraction that binds to P making it unavailable. Beneficial microbes, such as P solubilizing microorganisms can increase the available P in soil and improve plant growth and productivity. In this study, we evaluated the effects of environmental conditions (climate, soil parameters), plant genotype, and level of plant association (rhizosphere or endophytic root organism) on the abundance and diversity of phosphorus solubilizing microorganisms in a *Salix* production system. We hypothesized that a lower number of endophytic fungi may possess the ability to solubilize P compared to the number of rhizosphere fungi with the same ability. We also expect that the plant genotype and the experimental site with its environmental conditions will influence fungal diversity. Two *Salix* genotypes grown in pure and mixed cultures were investigated for their fungal microbiome community and diversity in the rhizosphere and endosphere during two growing seasons. We found that the rhizosphere fungal community was more diverse. A general dominance of Ascomycota (*Dothideomycetes*) and Basidiomycota (*Tremellomycetes*) was observed. The classes *Agaricomycetes* and *Pezizomycetes* were more frequent in the endosphere, while *Tremellomycetes* and *Mortierellomycetes* were more abundant in the rhizosphere. Plot-specific soil properties (pH, total organic carbon, and nitrogen) significantly influenced the fungal community structure. Among the culturable fungal diversities, 10 strains of phosphate solubilizing fungi (PSFs) from roots and 12 strains from rhizosphere soil were identified using selective media supplemented with di-calcium and tri-calcium phosphates. The fungal density and the number of PSF were much higher in the rhizosphere than in the endosphere. *Penicillium* was the dominant genus of PSF isolated from both sites; other less frequent genera of PSFs were *Alternaria, Cladosporium*, and *Clonostachys*. Overall the main factors controlling the fungal communities (endophytic vs. rhizosphere fungi) were the soil properties and level of plant association, while no significant influence of growing season was observed. Differences between *Salix* genotypes were observed for culturable fungal diversity, while in metagenomic data analysis, only the class *Dothideomycetes* showed a significant effect from the plant genotype.

## Introduction

Phosphorus (P) is an essential plant nutrient provided by a non-renewable resource (Filippelli, 2008; Shen et al., 2011). Fluoroapatite is the main and non-renevable resource from which fertilizers are produced (Filippelli, 2008). Moreover, P compounds released during weathering are usually complex and are not immediately bio-available (Filippelli, 2008; Singh and Satyanarayana, 2011). Plants and microorganisms release extracellular phosphatases from roots and recover orthophosphate ions from mineralizing phosphor-organic compounds (Richardson and Simpson, 2011). Furthermore, P solubilization is a microbial-driven process of hydrolysis of organic and inorganic P compounds to simpler compounds that can be utilized by plants (Richardson and Simpson, 2011).

Phosphorus solubilizing microorganisms can increase the availability of P for plants from the soil P pool, reducing the need for P fertilization. More P solubilizing bacteria have been reported than P solubilizing fungi (PSFs), but the latter are more effective, as PSFs do not lose the ability to solubilize P after subculture (Zhang et al., 2011). There are reports of various PSF species, such as *Aspergillus* spp., *Penicillium* spp., *Trichoderma viride*, *Arthrobotrys oligospora*, *Cephalosporium* sp., or *Cladosporium* sp. (Khan et al., 2010; Patil et al., 2012; Sharma et al., 2012; Ram et al., 2015; Li et al., 2016; Alori et al., 2017). In certain cases, these fungi may form symbioses with plants, develop mycelial networks, produce plant growth-promoting metabolites, and increase plant P and nitrogen uptake from the soil, e.g., by increasing root surface area (Baum et al., 2009). Chatli et al. (2008) isolated PSFs belonging to the genera *Aspergillus* and *Penicillium* from the rhizosphere of willow (*Salix alba* L.) growing in the *trans*-Himalayan region. Few experiments have demonstrated that PSFs isolated from other host plants could be used to increase yields of tomato, maize, and wheat (Khan and Khan, 2002; Reyes et al., 2002; Sharma et al., 2012; Ram et al., 2015).

The European Union directive (April 2009) on the promotion of the use of energy from renewable sources (Directive 2009/28/EC) states that 20% of the total energy in Europe should be generated from renewable sources^1^. Generally modern agriculture is focused mostly on reaching high yields by the use of best-performing plant species grown in pure culture. Such pure cultures are often easier in maintenance than mixed cultures, but pure cultures have been shown to be more vulnerable to pests and diseases in many cases. Thus, the introduction of mixed genotypes plantations could limit the losses due to pests and diseases while significantly enhancing biodiversity (Hoeber et al., 2018; Schweier et al., 2019). This may improve the sustainability of biomass production on SRCs but our knowledge in this area is still limited. Additionally selection of tree species for SRC is critical and depends on the local climate and soil conditions. SRCs are generally confined to fast growing tree species, mainly from the genera *Populus*, *Salix*, *Eucalyptus*, and *Robinia* (Navarro et al., 2016). *Salix* species are fast growing trees that possess high economic value because of their high potential to contribute to renewable energy generation in Europe (Sevel et al., 2012; Weih et al., 2021). This woody crop can be planted on soils that are less suitable for farming of food crops and can be fertilized with sewage sludge, wastewater, or ashes which contain high amounts of nitrogen and phosphorus (Dimitriou and Aronsson, 2011). Many species of *Salix* can control P uptake and metabolism, although the corresponding mechanisms are still largely unknown (Rennenberg and Herschbach, 2013).

Rhizosphere and endophytic fungi play important roles in plant growth and development. Their ecology and function is different and depends on various parameters (Hrynkiewicz and Baum, 2012). The level of plant association may be affected by soil properties, climate, weather conditions, and host plant genotype. Likewise, understanding how the willow plantations in the form of monoculture and mixed genotypes could affect the overall microbial diversity is key information. This study bridges the gap by providing knowledge on the rhizosphere and endophytic fungal diversity in willow SRCs. Hence, the main aim of this research was to investigate the diversity of rhizosphere and endophytic fungi from two willow species, *S. dasyclados* (cultivar “Loden”) and *S. schwerinii* × *S. viminalis* (“Tora”), as well as their mixture at two sites located in Germany and Sweden. The two species selected for the study are phenotypically different, and “Tora” is known for its high productivity compared to “Loden” (Hoeber et al., 2017). The mixture of two host species may result in increased diversity of fungi compared to growing the same species in pure culture. The two investigated experimental sites represent ECOLINK-Salix within a global tree diversity network (Verheyen et al., 2016). The two selected sites are similar in terms of planting time, design, and management (e.g., fertilization, timing of shoot harvests) but vary by local climate and soil conditions. This might provide more information about importance of climate and soil nutrient content on willow microbiome. We evaluated the effect of climate, soil conditions, level of plant association, and planting design on the abundance and diversity of PSFs by applying culture-dependent and culture-independent (metagenomic) methods. Using culture-dependent techniques is critical as it allows application of this study for future research in using these strains as inoculants in plants. Moreover, culture-independent methods give more insight into the culturable and unculturable fungal diversity present in the two experimental sites. In addition, to assess the potential impact of PSFs on the P supply of *Salix* in fall, we tested the P solubilization efficiency of fungal isolates. We hypothesized that a lower number of endophytic fungi may possess the ability to solubilize P compared to the number of rhizosphere fungi with the same ability. Second, the site-specific climate and soil conditions and the *Salix* species genotypes may determine the abundance and diversity of total fungi and PSFs.

## Materials and Methods

### Site Description and Sampling

The two experimental sites have been well-established SRCs since spring 2014 (Hoeber et al., 2018). The first experimental site is located in Uppsala, Sweden (59°49′13.4″N, 17°38′25.2″E), and the second is located in Rostock, Germany (54°03′41.0″N, 12°04′54.7″E). Both sites are former arable fields. The experimental site in Uppsala is located on fine-textured mineral soils, mainly Vertic Cambisols (according to IUSS Working Group WRB, 2015). The experimental site Rostock is dominated by Stagnic Cambisols developed from loamy sands.

The study sites vary by local climate and soil conditions. According to the Köppen–Geiger classification, the climate in Uppsala is boreal (Dfb: snow, fully humid with warm summers; Kottek et al., 2006). The average annual rainfall is 551 mm, and the average annual air temperature is 5.8°C (1970–2000; http://www.worldclim.org/current). Winters are usually not as cold as in other cities at similar latitudes due to the influence of the Gulf Stream. Rostock is situated on the Baltic coast in a warm temperate climate (Cfb: fully humid with warm summers). The average air temperature of Rostock is higher than that of Uppsala (8.4°C), but the rainfall sum is similar (600 mm).

The meteorological data obtained from https://www.worldweatheronline.com/showed similar trends in the 2018–2019 period for both studied localities. In general, these years were warmer than average (2009–2019). However, lower than average monthly temperatures were recorded in February (Rostock and Uppsala) and March (Rostock) 2018. The interannual variability in the monthly distribution of rainfall was high. The 2018 year was significantly drier and the 2019 year was much wetter than average (2009–2019; Supplementary Table 1 and Supplementary Figure 1). Two *Salix* genotypes, “Loden” (*S. dasyclados*) [L] and “Tora” (*S. schwerinii* × *S. viminalis)* [T], were cultivated as pure cultures and mixtures [LT] at the two sites. The L genotype is characterized by shorter shoots and a larger leaf area than the T genotype (Hoeber et al., 2018).

**FIGURE 1:**
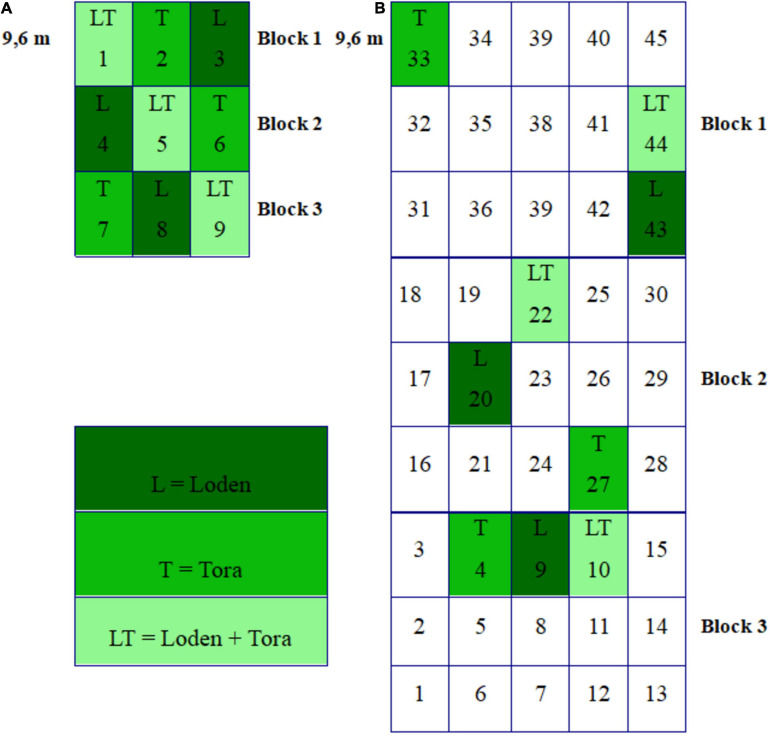
**(A)** The randomized block design of the ECOLINK-Salix field trial in Germany (Rostock) consisting of 9 plots (each 9.6 m × 9.6 m) in three blocks with genotype monocultures and mixtures. **(B)** The randomized block design of the ECOLINK-Salix field trial in Sweden (Uppsala) consisting of 45 plots (each 9.6 m × 9.6 m) in three blocks with genotype monocultures and mixtures.

Willow roots and soils were sampled from the two experimental sites during two seasons: Fall 2018 (Sweden – October 23rd, Germany – October 27th) and spring 2019 (Sweden – May 15th, Germany – May 18th). Both experimental sites were organized into three blocks, and the density of plants was 15,600 ha^–1^. Blocks were divided into plots randomly planted with different *Salix* genotypes (Figure 1) and their mixtures in all possible combinations. Treatments L, T, and LT were selected for investigation since they were present at both experimental sites (Figure 1). There were three replicates per treatment (nine plots per experimental site, in total), and each plot was 9.6 m × 9.6 m.

Three samples per plot (81 from Sweden and 81 from Germany per season, 324 in total) were taken by digging root samples with adhering soil (15 cm × 15 cm × 15 cm) at a distance of 6 m from each other for microbiological analysis. The organic litter layer (up to 5 cm thick) was removed and topsoil (0–25 cm) was sampled. Samples were carefully transferred to collection bags and covered with a thin layer of soil to prevent drying. Collected samples were immediately transported to the laboratory and used for analyses.

### Soil Physicochemical Analysis

Air-dried soil samples were passed through a 2 mm sieve. The total organic carbon (TOC) and total nitrogen (TN) contents were measured after dry combustion using a CHNS Vario Macro Cube elemental analyzer. Available phosphorus (P_av_) in 1% citric acid (van Reeuwijk, 2002) was determined by a spectrophotometric method using spectrophotometer UV–Vis Rayleigh UV-1601 (van Reeuwijk, 2002), and the pH (in H_2_O and 1M KCl) at 1:2.5 soil to solution ratio was determined by the potentiometric method using an Elmetron CP-105 pH-meter. Table 1 shows differences in soil parameters on both sampling sites and two growing seasons.

**TABLE 1:** Characteristics of rhizosphere soils.

Site	Season	Genotype	TOC (%)	TN (%)	C:N	P_av_ (mg⋅kg^–1^)	pH(H_2_O)	pH(KCl)
Sweden	Fall	L	1.69 ± 0.24*	0.16 ± 0.02*	10.3 ± 0.5b	101 ± 12.2b	5.7 ± 0.2b	5.3 ± 0.1
		T	1.59 ± 0.21*	0.16 ± 0.01*	10.2 ± 0.7	94.2 ± 13.6*	5.7 ± 0.1b	5.2 ± 0.2
		LT	1.65 ± 0.2*	0.16 ± 0.02*	10.4 ± 0.6	105 ± 28.1b*	5.7 ± 0.1	5.1 ± 0.1
	Spring	L	1.89 ± 0.29*	0.17 ± 0.02*	11.1 ± 0.5Aa	75.7 ± 20.1Ba*	6.0 ± 0.2Ba	5.2 ± 0.2
		T	1.72 ± 0.22*	0.17 ± 0.02*	10.2 ± 0.6B	93.6 ± 14.7A*	6.2 ± 0.2Ba	5.2 ± 0.1
		LT	1.63 ± 0.18	0.16 ± 0.02*	10.3 ± 0.5B	63.1 ± 19.8Ba	5.7 ± 0.2A	5.2 ± 0.1
Germany	Fall	L	1.21 ± 0.17	0.11 ± 0.02	11.3 ± 0.7*	101 ± 22.0Aa	6.0 ± 0.4*	5.8 ± 0.4*
		T	1.13 ± 0.13b	0.1 ± 0.01b	11.3 ± 0.7*	57.0 ± 7.3B	6.1 ± 0.3*	6.0 ± 0.3*
		LT	1.22 ± 0.21b	0.1 ± 0.01b	11.7 ± 1.0*	55.0 ± 12.3B	6.0 ± 0.3b*	5.7 ± 0.4*
	Spring	L	1.24 ± 0.2	0.11 ± 0.01	11.3 ± 0.9	50.5 ± 9.5b	6.2 ± 0.3*	6.1 ± 0.3*
		T	1.4 ± 0.24a	0.11 ± 0.01a	12.5 ± 1.3*	54.8 ± 11.3	6.3 ± 0.2*	6.2 ± 0.2*
		LT	1.49 ± 0.19a	0.12 ± 0.01a	12.4 ± 0.8*	57.3 ± 11.2	6.2 ± 0.2a*	6.1 ± 0.3*

At each sampling site (Sweden and Germany), three plots per three blocks for each variant of the experiment (Loden, Tora, and mixture of both genotypes) were sampled. In total, 108 root and soil samples from both samplings were collected. Soil properties were compared by site, season, and genotype (plots: L-Loden, T-Tora, and LT-mixture). Values are means ± SDs (*n* = 9). The significant differences with *p* < 0.05 are marked by the following symbols: * – differences between sites in the same season, small letters – differences between seasons within one site, and capital letters – differences between genotypes on the same site and season.

### Total Cultivable Fungal Density

Samples from fall 2018 were processed for cultivable fungal density and phosphate solubilizationability. The willow roots were carefully separated from the adhering rhizosphere soil. One and a half grams of roots were surface sterilized with 60% alcohol (3 min) by vigorous shaking and then washed three times in sterile 2% NaCl solution. The roots were washed in sterile 5% H_2_O_2_ solution (10 min) and rinsed three times in sterile 2% NaCl solution. The last wash of 2% NaCl was plated on R2A medium (BD Difco, United States; sterilization control). The surface sterilized roots (1 g) were homogenized using a sterile mortar and pestle under sterile conditions and then transferred to Falcon tubes containing 9 ml of sterile 0.5% NaCl solution. For the rhizosphere soil samples (from uppermost mineral part of soil affected by the plant roots), 1 g was transferred to Falcon tubes with 9 ml of sterile distilled water, and dilutions were performed.

For the total fungal density, potato dextrose agar (PDA) medium (BD Difco, United States) supplemented with tetracycline at 100 mg/l was used. Serial dilutions of 10^–2^ and 10^–3^ of the root samples and 10^–3^ and 10^–4^ of the rhizosphere soil were selected for spread plating. Plates were kept at 24°C, and the total number of fungal colonies on PDA medium was counted after day 7 and presented as colony forming units (c.f.u).

### Screening PSFs From Roots and Rhizosphere Soil

The isolation and selection of PSFs were performed on three selective media (NBRIP, PVK; Nautiyal, 1999, and DCP; modified Pikovskaya, 1948; Supplementary Table 2) containing either tri- (NVRIP, PVK) or diphosphates (DCP). In total, 108 plates per medium were used to evaluate P solubilizing and non-PSF.

The serial dilutions from the roots and the rhizosphere soil (100 μl each) were spread plated on the three selective media. For NBRIP and PVK media, 10^–5^–10^–6^ dilutions were used for both types of samples. For DCP medium, 10^–3^–10^–4^ dilutions were used for the roots, and 10^–4^–10^–5^ dilutions were used for the rhizosphere soil samples. Three technical replicates were prepared for each sample. Plates were kept at 24°C for 7 days and observed for fungal halos indicated phosphorous solubilization. Calculations were performed to determine the number of PSF and the total number of fungi growing on each of the three media. All PSF isolates unique to each plot were subjected to molecular identification. The fungal density was determined by the log_10_ [c.f.u. (g f.w. soil)^–1^ or (g f.w. roots)^–1^] values for both the total fungal count and those screened with selective media.

### Molecular Identification of Fungal Strains

Twenty-two selected fungal isolates (12 from Germany and 10 from Sweden) were cultivated on fresh PDA medium (BD Difco, United States). Fungal DNA was isolated from fresh mycelium using the Plant and Fungi DNA Purification Kit (Eurx, Poland). The concentration of DNA was measured using a UV–Vis spectrophotometer (Thermo Scientific NanoDrop2000, United States). The ITS region was amplified using ITS1 (5-CTTGGTCATTTAGAGGAAGTAA-3) and ITS4 (5-TCCTCCGCTTATTGATATGC-3) primers (Martin and Rygiewicz, 2005; Manter and Vivanco, 2007). PCR clean-up was carried out using a PCR/DNA Clean-Up Purification Kit (EurX, Poland). The presence of ITS sequences was confirmed on a 1% agarose gel (1X TBE buffer) with the addition of Simply Safe dye (EurX, Poland). Samples were sent for sequencing to the Institute of Biochemistry and Biophysics PAS^2^. Contigs were assembled using Sequencher 5.4.6 software and compared with the NCBI database using BLASTn^3^ to find sequences that showed the highest similarity to the assembled contigs. The DNA sequence generated for this study were deposited in the NCBI GenBank under the following accession numbers: MW342736–MW342757 (as shown in Supplementary Table 3). The phylogenetic tree was constructed using the NJ method in MEGA 7, and 1,000 bootstrap replicates were used to assess branching support (Tamura et al., 2013; Kumar et al., 2016). The *p*-distance method was calculated (Saitou and Nei, 1987). The phylogenetic tree was visualized using Interactive Tree of Life (iTOL) v3 (Letunic and Bork, 2016).

### Metagenomic Analysis

Total DNA was extracted from 50 mg of lyophilized willow roots and rhizosphere soil samples using Plant and Fungi DNA Purification Kit (EURx, Poland) according to the manufacturer’s protocol. Three biological replicates were prepared for each plot. The amount of isolated DNA was quantified fluorometrically (InvitrogeneQubit 2.0, United States) and the quality was assessed spectrophotometrically (Thermo Scientific NanoDrop 2000, United States) and the preparations were diluted to 1 ng/μl.

Fungal ITS amplicon libraries were generated in two-step PCR, as described by Thiem et al. (2018) using fungal primers (uITS1 and uITS2) then with M13 and M13R primers with P5/P7 adapters and barcodes (different MID sequences for each sample). Libraries were purified twice with Agencourt AMPure XP (Beckman Coulter) according to the manufacturer’s protocol. The quality of the pooled libraries was assessed on a Bioanalyzer chip (Agilent) and they were quantified with KAPA Library Quantification Kit for Illumina Platform using LightCycler 480 (Roche) according to the manufacturers’ protocols. The final pool was diluted to 4 nM, denaturated, mixed with 5% of PhiX control library and sequenced with the use of 2 × 300 cycles kit v.3 on a MiSeq machine (Illumina).

The resulting read pairs were denoised with dada2 (Callahan et al., 2016) and the fungal sequences were processed with ITSx (Bengtsson-Palme et al., 2013), and all fungal ITS1 sequences were used in the downstream analyzes. The reads were de-replicated and OTUs were constructed using vsearch (Rognes et al., 2016) at 0.03 dissimilarity level, then singletons as well as doubletons (OTUs consisting of one or two sequences only) were removed. The sequences were classified with naive Bayesian classifier (minimum 80% bootstrap support was required; Wang et al., 2003) using ITS1 extracted from UNITE v.7 (fungi), and the non-fungal sequences were removed. The final data were sub-sampled to 300 (fungi) sequences per sample 20 times, sequences names were mangled to reflect the iteration, the sets were pooled, de-replicated, and OTUs were constructed as described earlier. OTU tables were then averaged over the 20 subsamples and the entries were rounded to the nearest integer with a Perl script to yield the final tables.

### Data Analysis

**FIGURE 2:**
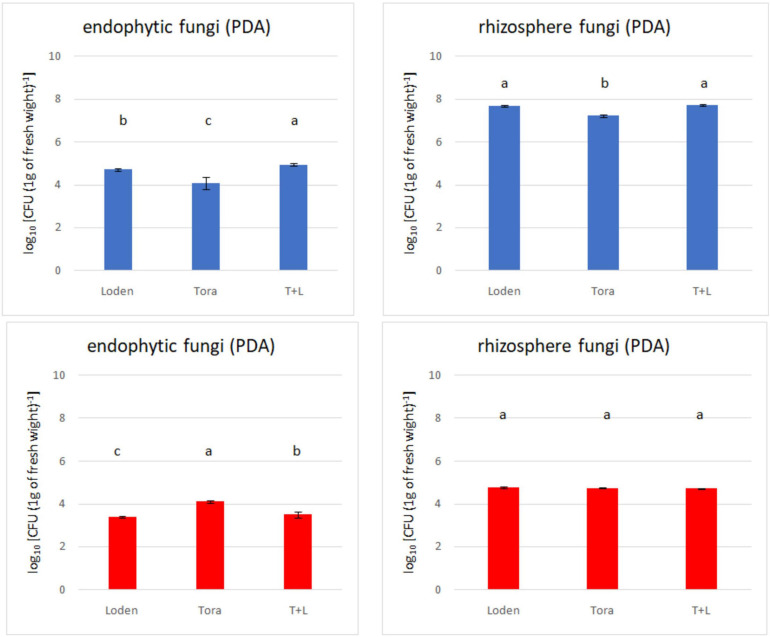
Total fungal count (endophytes and rhizosphere) from two experimental sites (Sweden and Germany) in fall 2018. The upper panels (blue bars) indicate the total fungal counts for Swedish samples; the lower panels (red bars) indicate the total fungal counts for Germany samples.

**FIGURE 3:**
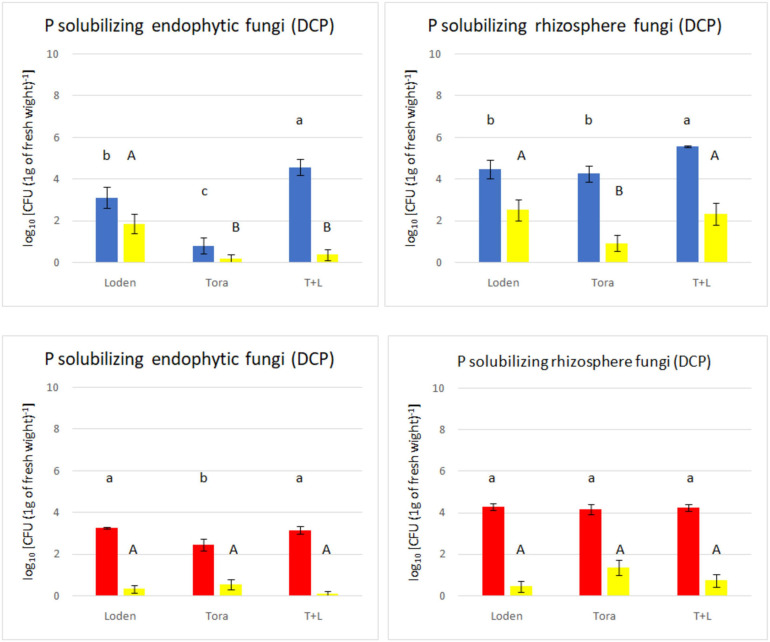
Total non-P solubilizing and P solubilizing fungi count (endophytes and rhizosphere) from two experimental sites (Sweden and Germany) in fall 2018. The upper panels (blue bars) indicate the total fungal counts from selective media for Swedish samples; the lower panels (red bars) indicate the total fungal counts from selective media for Germany samples. The yellow bars indicate the number of phosphate solubilizing fungi found among the non-P solubilizing fungi.

Statistical analysis for screening of PSFs was performed using the Statistica software package (version 13.0, StatSoft) based on three replicates for each sample variant. For total density of culturable strains (Figure 2) and PSF screening (Figure 3) nine replicates (three samples from each of three plots) for each genotype present on site were used. Normality was tested using Shapiro–Wilk test and homogeneity of variance using Levene’s test. Samples that were outside of two standard deviations range from mean were removed. The one-way analysis of variance (ANOVA) was used to determine whether there are any statistically significant differences between the means of total fungi count for each genotype on PDA and selective media and PSF count for genotypes for each experimental site. Upper case letters represent significant differences for PSF count for genotypes for each experimental site (Figures 2, 3).

For metagenomic data analysis, Bray–Curtis distance matrices based on Wisconsin double-standardized OTU tables were calculated with vegdist in R. Non-metric multidimensional scaling (NMDS) and canonical correspondence analysis (CCA) analyzes were performed within R with vegan’s metaMDS and cca functions. In case of CCA, forward selection procedure implemented in ordistep was used for stepwise model building. Significance of differences between sample clusters was assessed with ANOSIM and PERMANOVA in vegan’s anosim and adonis functions, respectively. *P*-values < 0.05 was considered significant. Variance partitioning was performed with the varpart function. Significance of differences in means (number of observed OTUs, Shannon’s H′, Shannon’s E, taxa, and functional groups distribution) was assessed with ANOVA with *post hoc* Tukey’s HSD analysis, unless assumptions of normality of data and/or homogeneity of variance were violated, in which case robust ANOVA implemented in raov of the Rfit package was used to check for general *p*-value. All figures were plotted with standard R graphic functions.

## Results

### Soil Properties and Climatic Conditions at the Two Experimental Sites

As shown in Table 1, in Uppsala the rhizosphere soil parameters TOC, TN, C:N was higher while P_av_, pH(H_2_O), pH(KCl) was lower. ANOVA analysis revealed that the soil properties differed significantly between the experimental sites (Table 1). Soil samples from Sweden had higher contents of TOC, TN, and P_*av*,_ while soil samples from Germany were characterized by higher C:N ratios and pH values. Soil parameters at both experimental sites differed slightly in spring. Significant differences between genotypes were observed for the TOC (T and LT in Germany), pH (all plots in Sweden) and P_av_ content, which were higher in fall (L and LT plots in Sweden and L plots in Germany; Table 1).

### Identification of Dominant PSFs and Total Fungal Density in Willow Genotypes and Mixtures

The overall density of cultivable fungi ranged from 3.38 to 4.94 log10 [measured as colony-forming units: c.f.u. (g d.w. roots)^–1^] for endophytes from both experimental sites. In the rhizosphere soil, the total fungal count ranged from 4.71 to 7.72 log10 [c.f.u. (g d.w. soil)^–1^]. Significant differences among the analyzed willow genotypes were observed for endophytic fungal density (Figure 2), but this was not observed for rhizosphere fungi. The endophytic fungal density was lower than that of rhizosphere fungi. The difference in fungal density between the endophytes and rhizosphere soil was significantly greater in Swedish samples than German ones. Generally, the highest fungal density was recorded in LT samples from Sweden and T samples from Germany.

The medium supplemented with triphosphate (PVK) showed nofungal growth, and therefore, it was excluded from the analysis. The total number of culturable endophytic fungi ranged from 0.80 to 4.57 log10 c.f.u g^–1^, among which PSF ranged from 0.12 to 1.86 log10 c.f.u. g^–1^ but showed no significant differences with the exception of Loden (only in Sweden). The endophytic fungal density was lower than that of rhizosphere fungi, ranging from 3.38 to 4.94 log10 c.f.u. g^–1^, whereas rhizosphere fungi ranged from 4.75 to 7.72 log10 c.f.u. g^–1^. The LT genotype showed the highest non-PSF density at both experimental sites (Figure 3). The endophytic fungal density was lowest for the Tora genotype at both experimental sites. The phosphate solubilizing ability of fungi from the Swedish site (found mostly in rhizosphere soil) was higher than that in fungi from the German site.

**FIGURE 4:**
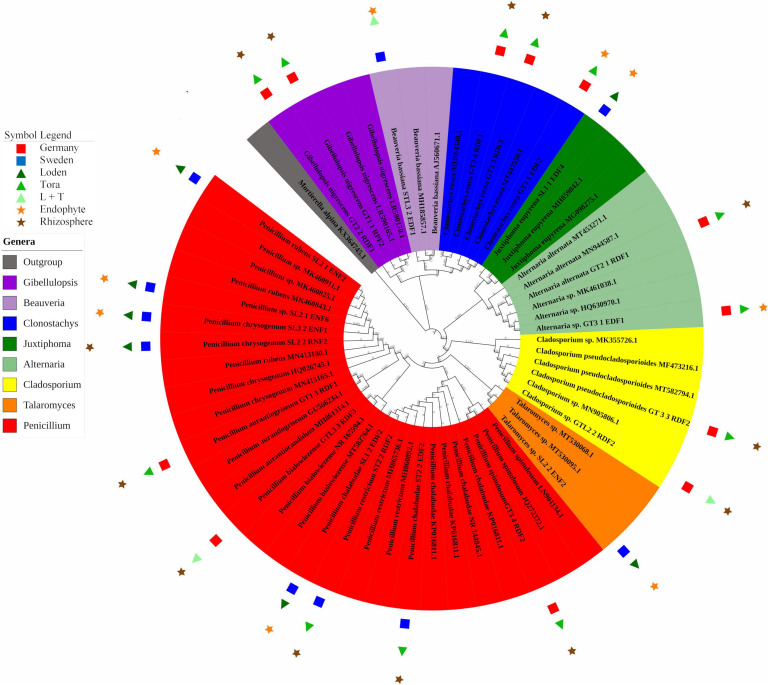
Phylogenetic analysis of culturable fungi isolated from three willow genotypes classified at the genus level. For details of isolates with their GenBank accession numbers, see Supplementary Table 3. Reference sequences with closest BLAST match were used (strains without symbols) to construct the phylogenetic tree. The 22 fungal strains with PSF activity are marked with their respective symbols (see legend on the left).

In total, 22 fungal strains were isolated from roots (10 strains) and rhizosphere soil (12 strains; Figure 4). *Clonostachys* and *Penicillium* were the dominant genera at the German site (30% for each), while at the Swedish site, *Penicillium* alone was the dominant genus (70%; Figure 4). Most of the strains found at the German site were isolated from the rhizosphere soil of T, in contrast to the Swedish site, where the majority was endophytic isolates from the L genotype. *Penicillium* was the only fungal genus occurring at both sites. The German site showed higher diversity in the rhizosphere, with five different fungal genera (*Penicillium*, *Clonostachys*, *Alternaria*, *Gibellulopsis*, and *Cladosporium*). For the Swedish site, the highest diversity was obtained for endophytes of the L genotype with three different species (*Penicillium*, *Talaromyces*, and *Juxtiphoma*). In total, 54% of the identified strains were isolated from T, 31% from L, and 13% from LT samples. The endophytes comprised 54% of the total number of identified fungi.

### Experimental Site and Level of Plant Association Shaped the Community Structure in Willow SRCs

**FIGURE 5:**
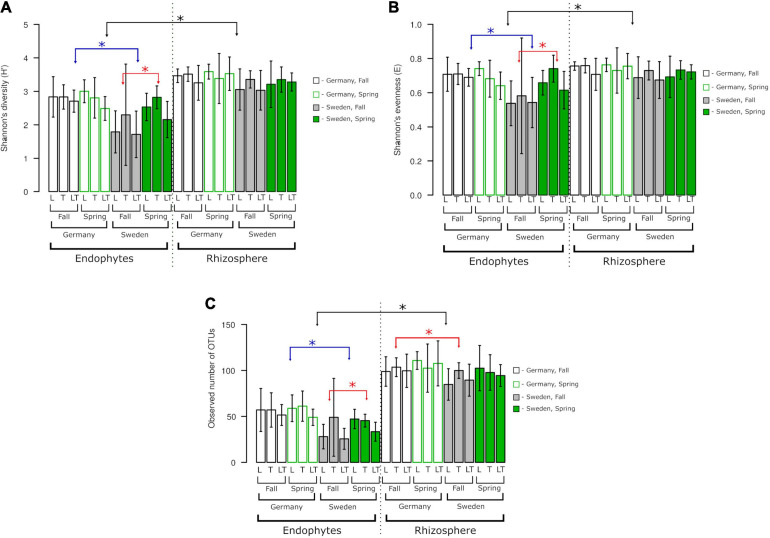
Fungal species richness, diversity, and evenness across two experimental sites, rhizosphere soil and roots. OTUs constructed at 0.03 dissimilarity for fungal sequences. **(A)** Shannon’s (H‘), **(B)** Shannon’s (E), **(C)** observed number of OTUs. Robust ANOVA with Tukey’s *post hoc* analysis was used to assess the significance of differences between experimental sites, rhizosphere soil, and roots. Colors denote the following: black – significant difference between endophytes/rhizosphere. ^∗^Indicates significant difference between variants indicated by arrows.

The alpha diversity of the fungal community was not influenced by genotype but by site or the level of fungi association with the plant (rhizosphere vs. endosphere; Figure 5). Diversity (H′), species richness (observed OTU number) and evenness (E) were higher in the rhizosphere than in the endosphere. Endophytic communities at the German site were more diverse and even harbored more OTUs than those at the Swedish site; however, there were no differences between seasons. At the Swedish site, alpha diversity was higher in spring than in fall. There were no differences between variants in rhizospheric communities.

The alpha diversity analysis did not show significant differences between genotypes. Overall, Shannon’s diversity (H′), Shannon’s evenness, and observed OTUs revealed significant differences between the endophytes and rhizosphere fungal diversity regardless of the experimental site and seasons. The endophytic diversity at the two experimental sites in Germany and Sweden was significantly different from that of the rhizosphere fungi. A significant effect of seasonality was observed only for endophytes from Sweden (Figure 5). The number of observed OTUs for rhizosphere fungal diversity showed a greater tendency than that of the endophytes, and this difference was prominent between the experimental sites in Germany and Sweden during the fall.

**FIGURE 6:**
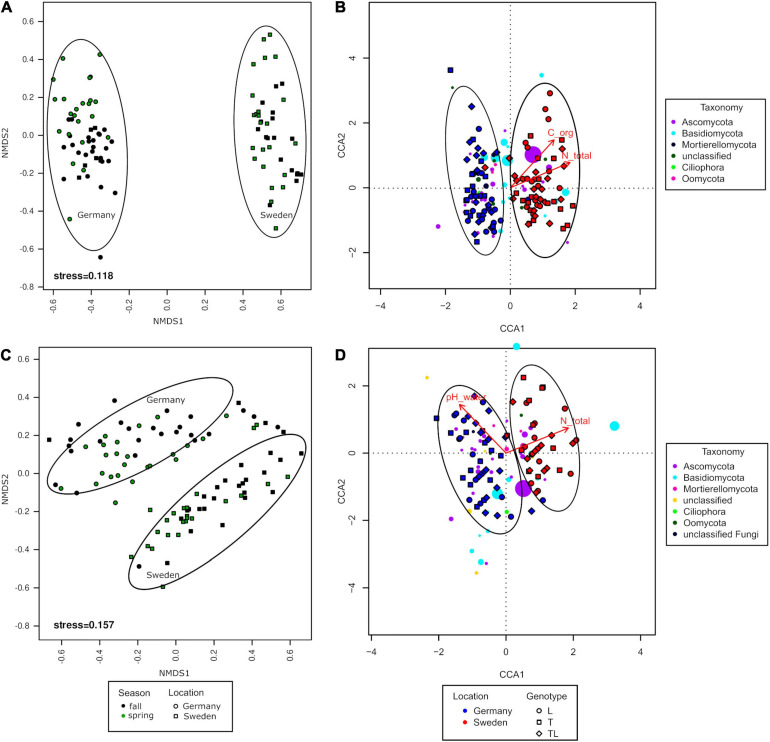
Analysis of log-transformed and Wisconsin double-standardized Bray-Curtis dissimilarity matrix for rhizosphere soil and root fungal communities associated with *Salix* genotypes L (Loden), T (Tora), and LT (mixture) between two seasons (fall, spring) and two sampling sites (Germany and Sweden). **(A,C)** NMDS (non-metric multidimensional scaling analysis); **(B,D)** CCA (canonical correspondence analysis).

The NMDS analysis revealed that the fungal communities clustered according to the experimental sites, but this was not observed for seasons and genotypes (Figure 6). The grouping was significant for roots (PERMANOVA, *F* = 0.5050, df = 1, and *P* = 0.0001) and for soil (PERMANOVA, *F* = 0.1830, df = 1, and *P* = 0.0001). The differences in variance were not significant for roots (PERMDISP, *P* = 0.579) or soil (PERMDISP, *P* = 0.2911). The distance between the fungal communities in the two experimental sites was significantly larger for the rhizosphere soil than for roots.

The CCA showed that the fungal communities were significantly different between the two experimental sites only (Figures 6B,D). In the rhizosphere soil, total organic C and total N were the two environmental variables shaping fungal diversity at the Swedish experimental site (Figure 6). TN explained 7.5% of variance whereas TOC explained 2.4%. In contrast, the fungal communities in the roots of the German experimental site were mostly influenced by pH(H_2_O), whereas at the Swedish experimental site, the most influential factor was TN (Figure 6). TN explained 2.5% of the variance, while pH(H_2_O) explained 1.5%.

In conjunction with the culturable fungal diversity, the fungal libraries from the rhizosphere soil and endophytic community showed significant dominance of the phylum Ascomycota, followed by Basidiomycota and Mortierellomycota (Supplementary Figure 2).

**FIGURE 7:**
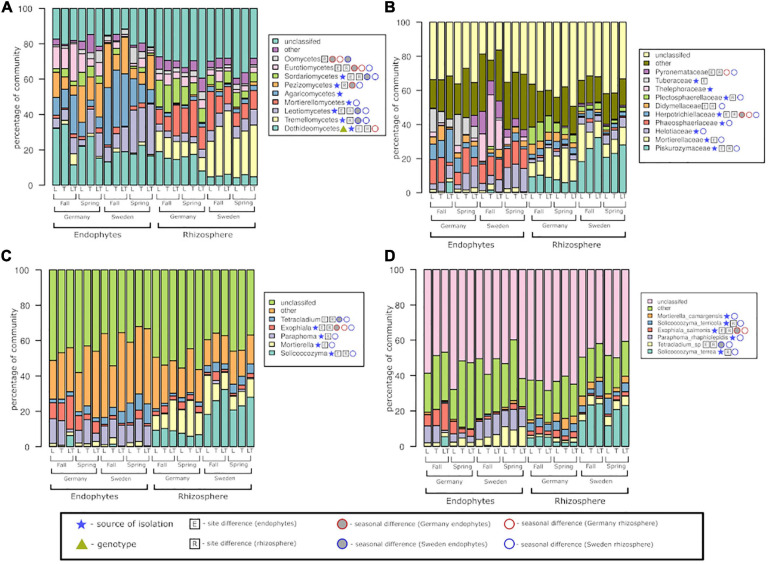
Fungal community structure at the level of class **(A)**, family **(B)**, genus **(C)**, and species **(D)** among rhizosphere soil and roots, two experimental sites, and two seasons.

At the class level, the fungal community was dominated by *Dothideomycetes* and *Leotiomycetes*, with significant differences seen for both endophytes and rhizosphere soil fungi. The above two classes were significantly different based on both the experimental sites and seasons as well (Figure 7). *Agaricomycetes* and *Pezizomycetes* showed high abundance and were exclusive to endophytes only. All classes except *Agaricomycetes* displayed significant differences between the two seasons. The rhizosphere soil fungal community was dominated by *Tremellomycetes* and *Mortierellomycetes*; the former showed significance among the two sites and seasons, while the latter showed only seasonal effects. *Dothideomycetes* was the only class exhibiting differences among genotypes.

At the family level, *Piskurozymaceae* showed greater fungal diversity among the rhizospheric fungi than the endophytes (Figure 7). *Helotiaceae* and *Phaeosphaeriaceae* were dominant and significantly represented in the Swedish site. *Tuberaceae* and *Herpotrichiellaceae* reads were found mostly in German samples, but the former did not show seasonal changes. Among the fungal endophytic communities, the effect of season was not observed for *Tuberaceae* and *Thelephoraceae.* In the rhizosphere fungal community, both sites were dominated by *Piskurozymaceae* and *Mortierellaceae*, mainly at the Swedish site. At the German site, the family *Plectosphaerellaceae* displayed a significant seasonal effect.

The endophytes *Paraphoma* and *Exophiala* were the most frequently occurring genera in both the experimental sites and seasons. The genus *Exophiala* was present in greater numbers at the German site than at the Swedish site; in contrast, the *Tetracladium* genus was present mostly at the Swedish site. On the other hand, the rhizosphere fungal libraries were dominated by reads of the genera *Solicoccozyma* and *Mortierella* mainly at the Swedish site. The rhizospheric fungi were significantly different in the two seasons and were more prominent at the Swedish site than at the German site.

At the species level, the endophytic fungal community consisted mostly of *Tetracladium* sp., *Paraphoma rhaphiolepidis* and *Exophiala salmonis*, whereas most of the identified fungi from the rhizosphere soil belonged to *Salicoccozyma terrea* (with the highest occurrence at the Swedish site). Based on the experimental sites, the rhizosphere fungi at the German site showed significantly more fungal reads belonging to *E. salmonis*. *E. salmonis* was the only species to show significant differences between the endophytic and rhizospheric communities at the German site.

## Discussion

This study is first in describing the PSF in woody SRC and comparing the fungal diversity and community structure between sites, seasons, and genotypes. The beta diversity revealed that experimental site drives the fungal community structure. Abundance as well as alpha and beta-diversity of fungal community were mainly driven by TOC, TN, and pH. We found *Penicillium* to be the dominant genus of PSF in the group of isolated fungal strains, while this genus was not detected in the metagenomic analysis. This may be due to the presence of other abundant fungal genera that may mask its presence. The level of plant association (endophytic or rhizosphere fungi) was the main factor driving fungal diversity and community structure (number of observed OTUs was greater for rhizosphere fungi than endophytic fungi). Differences for seasons and genotypes were present but were not particularly prominent.

### Physico-Chemical Soil Properties and Climate Distinct at the Two Experimental Sites

To date, only a few studies have characterized the fungi associated with woody crops from different geographic locations, e.g., *S. alba* in India (Chatli et al., 2008), *Salix viminalis* in the United Kingdom (Barnes et al., 2018), *S. viminalis* and *S.* × *dasyclados* in Germany (Baum et al., 2006), and *S. viminalis* and *S. schwerinii* in Sweden (Hrynkiewicz and Baum, 2012). The soils in the investigated sites can be described as fertile and biologically active, with optimal properties for willow cultivation (Guidi et al., 2013). Soils at the Swedish site contained a higher content of TOC, TN, and P_av_ than soils at the German site, which likely resulted from primary soil properties related to their different pedogenesis (Vertic and Stagnic Cambisols) such as texture and nutrient abundance. However, the studied soils were moderately to slightly acidic, which undoubtedly favors the bioavailability of P (Yue et al., 2000; Richardson et al., 2009; Yadav et al., 2012). TN is frequently found to be a critical factor for fungal diversity (Allison et al., 2007; Lauber et al., 2008; Zhou et al., 2016). The available P in soil may influence fungal communities in the rhizosphere to some extent (He et al., 2016; Rosenstock et al., 2016; Williams et al., 2017). In our case, the Swedish site had significantly higher P_*av*,_ but we observed no significant influence of this variable. This confirms N-limited rather than P-limited conditions, as is common at most arable sites (Williams et al., 2017).

A statistically significant short-term seasonal increase in TOC was recorded at the experimental site in Germany, but a similar trend was also observed at the experimental site in Sweden. This can be attributed to the leaf and root litter inputs in combination with no-till management. However, research by Hoeber et al. (2020) showed that climatic factors may significantly determine the rate of decomposition of leaf litter. These authors found that the decomposition rates in Germany were 43% faster than those in Sweden. The fine roots of willows can also be an important source of soil organic matter (Kahle et al., 2007). A slight decrease in P_av_ concentrations in spring suggested nutrient depletion. No effects of nutrient depletion on the yields were observed in Sweden. These results were supported by the findings of (Kahle et al., 2007).

### Dominating PSF Identity and Total Fungal Density in Willows

Among the culturable diversities, the number of PSF was much higher in the rhizosphere fungi than in the endophytic fungi. This supports our hypothesis that lower number of endophytic fungi may possess ability to solubilize P than rhizosphere fungi. We found *Penicillium* to be the dominant genus of PSF isolates from the SRCs; other, less frequent isolates were of the genera *Alternaria, Cladosporium*, and *Clonostachys*. Similarly, Chatli et al. (2008) reported members of *Penicillium* to be among the dominant strains isolated from woody crop species. These strains were also abundant in the rhizosphere of *Salix* spp. in Lithuania (Repečkienė et al., 2009). The genus *Clonostachys* (isolated from the rhizosphere and endosphere) was specific to the German site, whereas *Juxtiphoma*, *Talaromyces*, and *Beauveria* were specific to the Swedish site. Apart from PSFs, we isolated non-PSF that were able to grow on selective media (NBRIP, PVK DCP). These fungi probably require low P concentrations to support growth. At the Swedish site, the fungal density in the three genotypes was found to range as follows: LT > Loden > Tora, in contrast to the German site, where it ranged as follows: Loden > LT > Tora. This effect might be based on the varying site-specific environmental conditions and their interactions with the genotypes.

*Penicillium* is commonly found as a PSF taxon (Chatli et al., 2008; Patil et al., 2012; Sharma et al., 2012). *Penicillium bilaiae* is even sold by NovoZymes as a bioinoculant enabling soil P mobilization. Levels of plant growth-promoting effects by *Penicillium* species were associated with increased uptake of P into shoots (Qiao et al., 2019). Although P solubilization ability is common in the genus *Penicillium*, various species and strains differ in their capacity to mobilize P due to differences in the secretion of organic acids, phosphatases, and phytase or in the operation of other P solubilizing mechanisms. The genus *Clonostachys* (isolated both from the endosphere and rhizosphere) was previously reported as an endophyte in *Salix* species growing in SRCs (Hosseini-Nasabnia et al., 2016). *Clonostachys rosea* is reported to be a mycoparasite in *Theobroma gileri* and as a biocontrol agent against *Phytophthora palmivora* and *Moniliophthora roreri* (ten Hoopen et al., 2003). To date, reports on the P solubilizing abilities of *Alternaria* and *Cladosporium* are scarce. There is not much information about PSF isolates from other woody plants. Schmidt et al. (2018) investigated endophytic community of poplar, species that is also commonly grown as SRCs but did not found any PSFs. Mishra et al. (2014) reported presence of several phosphorus solubilizing fungi in banana tree including *Fusarium* sp., *Trichoderma* sp. and present in this study *Penicillium* sp. In forest environment *Aspergillus* sp., *Cladosporium* sp., *Curvularia* sp. and several *Penicillium* species are frequently reported to possess high potential in P solubilization (Manoharachary and Nagaraju, 2017). There is several studies that use *Penicillium* species as bioinoculants that improve P availability for various plants (Omar, 1997; Ram et al., 2015; Yin et al., 2015; Li et al., 2016).

### Multiple Factors Influencing Alpha Diversity and Fungal Community Structure in Willow SRCs

The rhizosphere fungal community was more diverse in culture independent (metabarcoding) analyses, suggesting strong selective pressure in the interior of willow roots. Similar observations are common both for bacterial (e.g., Kielak et al., 2008; Bulgarelli et al., 2012) and fungal (e.g., Hrynkiewicz et al., 2012; Yergeau et al., 2015; Thiem et al., 2018; Furtado et al., 2019) communities associated with various plants. The reason for this phenomenon could be that roots may act as a filter or selection barrier for fungal species present in the rhizosphere soil, which can result in a lower number of endophytes (Garbeva et al., 2004; Lauber et al., 2008). This might be especially valid for woody roots such as those of *Salix* spp.

The classes *Agaricomycetes* and *Pezizomycetes* were more frequent in the endosphere, while *Tremellomycetes* and *Mortierellomycetes* were more abundant in the rhizosphere. These facts could be explained by the former two classes comprising mostly ectomycorrhizal fungi (Li et al., 2018), whereas the latter contains mostly saprophytic organisms (Francioli et al., 2020). Yergeau et al. (2015) reported the dominance of the class *Dothideomycetes* in willows, and the same was observed in our study. Members of this class are mainly endophytes and epiphytes and can be lichenized or lichenicolous fungi (Schoch et al., 2009). Moreover, *Dothideomycetes* was the only fungal class whose abundance differed between genotypes. *Agaricomycetes* was the only class whose abundance did not differ between seasons, which was caused by large differences within variants (i.e., high standard deviation). Similarly, reports by Shakya et al. (2013) from poplar showed no seasonal effect for rhizosphere fungi at any of their investigated sites.

At species level fungal community was mostly build from six species. For endophytes *Tetracladium* sp., *P. rhaphiolepidis* and *E. salmonis* were the most frequent. All three species are known grass endophytes while *E. salmonis* was additionally reported as an animal pathogen (Maciá-Vicente et al., 2016; Ricks and Koide, 2019; Gomzhina et al., 2020). Besides being pathogenic in animals it was previously isolated from the roots of poplar, which is another commonly grown tree species in SRCs (Maciá-Vicente et al., 2016). Most of the fungal reads from the rhizosphere soil belonged to *S. terrea*, but no information is available on this fungal species. The other *Solicoccozyma* species found in this study, *S. terricola* is a well known psychrotolerant yeast used in lipid production (Filippucci et al., 2016; Stosiek et al., 2019; Tasselli et al., 2019). Lastly, *Mortierella camargensis* isolated from grassland soils showed ability to produce arachidonic acid and lipase activity (Botha et al., 1999; Miklós et al., 2012).

The absence of the PSFs cultured from our samples in amplicon libraries is probably due to technical reasons. First, universal primers used to generate libraries might be biased against particular sequence variants (SVs), which together with SV scarcity in samples might result in excluding them from libraries.

Unconstrained ordination revealed that the level of community association with plants (rhizosphere vs. endosphere) and experimental sites were the two most important factors grouping the samples. This is expected, as (i) the difference between rhizosphere and endophytic communities is frequently observed (Thiem et al., 2018) and (ii) the sites differed in both climatic and soil conditions. Indeed, soil environmental parameters (TOC, TN, and pH) significantly influenced the fungal community structure. A significant influence of pH was unexpected, as it is usually not a limiting factor for fungi. Out of four factors we hypothesized would influence fungal communities in willow, level of community association with the plant, experimental site location and season turned out to exert a significant impact on fungi, while the effect of tree genotype was not as prominent. This fact can be explained by (i) the spatial distribution of fungal mycelium in soil, i.e., mycelium is able to freely grow out of particular field boundaries, effectively canceling actual differences, and (ii) genetic differences between analyzed genotypes may cause little effect on the soil fungal microbiome.

## Conclusion

The level of fungal community association with the plant (rhizosphere vs. root endophytes) is the most important factor shaping its diversity. The site, season, and planting design have a lower impact. The fungal diversity at the same level of plant association was mainly driven by soil properties such as TN, TOC, and pH. Among the culturable fungal diversities, *Penicillium* was dominant and commonly isolated genus as a P solubilizing taxon from both SRCs, while others less frequently isolated were the genera *Alternaria, Cladosporium*, and *Clonostachys*. Apart from PSFs, we isolated non-PSF that may require less P to support their growth. In general, a lower number of endophytic fungal strains possessed the ability to solubilize P compared to the number of rhizosphere fungal strains with this ability. The rhizosphere fungal community was generally more diverse than that in the endosphere at both willow SRC sites. This might suggest selective pressure on willow roots and emphasize the uniqueness of the fungal community. Fungal libraries of rhizosphere soil and endophytic communities showed significant dominance of the phyla Ascomycota followed by Basidiomycota and Mortierellomycota. The genus *Exophiala* was present in greater numbers at the German site, while the genus *Tetracladium* was present mostly at the Swedish site.

## Data Availability Statement

The datasets presented in this study can be found in online repositories. The names of the repository/repositories and accession number(s) can be found below: https://www.ncbi.nlm.nih.gov/bioproject, PRJNA716888; https://www.ncbi.nlm.nih.gov/genbank/, MW342736–MW342757.

## Author Contributions

PK participated in all analyses and wrote the first version of the manuscript. BF participated in preparation of manuscript. MG designed the bioinformatics pipeline, performed bioinformatics analyses, and participated in the preparation of the manuscript. PK and BF analyzed the results and the statistical output. PK, CB, and MW performed sampling at the locations, selected plant genotypes for the experiments, and provided input to the manuscript. PH did soil analyzes and participated in the preparation of the manuscript. KH designed and managed the field and lab experiments and participated in the preparation of the manuscript. All authors revised the manuscript and approve of its publication.

## Conflict of Interest

The authors declare that the research was conducted in the absence of any commercial or financial relationships that could be construed as a potential conflict of interest.

## Footnotes

1 https://ec.europa.eu/eurostat/web/energy/data/shares

2 http://oligo.ibb.waw.pl/

3 www.ncbi.nlm.nih.gov/BLAST

## Supplementary Material

Supplementary Figure 1 Average temperatures and average rainfalls in Rostock (Germany) and Uppsala (Sweden) in 2018 and 2019.

Supplementary Figure 2 Fungal community structure at the phylum level among rhizosphere soil and root samples, two experimental sites, and two seasons.

Supplementary Table 1 Meteorological data from two experimental sites in Sweden (SE) and Germany (GER). The maximal monthly temperature (max temp), minimal monthly temperature (min temp), average monthly temperature (avg temp), precipitation (rain), and monthly sunshine hours (sun) in 2018–2019. Data source: https://www.worldweatheronline.com.

Supplementary Table 2 Selective media (NBRIP, PVK, and DCP) used for the selection of phosphate solubilizing fungi.

Supplementary Table 3 Identified fungi with accession numbers. S – Sweden, G – Germany, L – Loden, T – Tora, LT – mixture, E – endophyte, and R – rhizosphere fungi.
