# Supplementary material for: The Effects of Host Plant Genotype and Environmental Conditions on Fungal Community Composition and Phosphorus Solubilization in Willow Short Rotation Coppice
Source: Front Plant Sci. 2021 Jul 5;12:647709. doi: 10.3389/fpls.2021.647709 (PMC8287252; doi:10.3389/fpls.2021.647709)

The effects of host plant genotype and environmental conditions on fungal community composition and phosphorus solubilization in willow short rotation coppice

Piotr Koczorski[1], Bliss Ursula Furtado[1], Marcin Gołębiewski[2,3], Piotr Hulisz[6], Christel Baum[4], Martin Weih[5],Katarzyna Hrynkiewicz[1*]

[1]Department of Microbiology, Faculty of Biological and Veterinary Sciences, Nicolaus Copernicus University, Torun, Poland

[2]Department of Plant Physiology and Biotechnology, Faculty of Biological and Veterinary Sciences, Nicolaus Copernicus University, Torun, Poland

[3]Interdisciplinary Center for Modern Technologies, Nicolaus Copernicus University, Torun, Poland

[4]Soil Science, Faculty of Agricultural and Environmental Sciences, University of Rostock, Rostock, Germany

[5]Department of Crop Production Ecology, Swedish University of Agricultural Sciences, Ullsväg 16, 75007, Uppsala, Sweden

[6]Department of Soil Science and Landscape Management, Faculty of Earth Sciences and Spatial Management, Nicolaus Copernicus University, Torun, Poland

Table 1. Meteorological data from two sampling sites in Sweden (SE) and Germany (GER). Maximal temperature in month (max temp), minimal temperature in month (min temp) average monthly temperature (avg temp), precipitation (rain) and hours of sun in month (sun) in 2018-2019. Data source: https://www.worldweatheronline.com/

**year**	2018																							
**month**	I		II		III		IV		V		VI		VII		VIII		IX		X		XI		XII	
**site**	SE	GER	SE	GER	SE	GER	SE	GER	SE	GER	SE	GER	SE	GER	SE	GER	SE	GER	SE	GER	SE	GER	SE	GER
**max temp [°C]**	0	4	-2	1	1	4	11	13	21	18	20	20	26	23	22	23	17	19	11	15	5	8	1	6
**min temp [°C]**	-3	1	-7	-3	-5	-2	3	6	9	10	10	13	15	16	13	16	10	12	5	9	2	4	-2	3
**avg temp [°C]**	-1	2	-4	-1	-2	1	6	9	15	14	16	16	20	19	17	19	13	15	8	12	3	6	0	4
**rain [mm/cm2]**	9.37	41.24	8.86	8.02	12.63	27.79	11.58	17.27	3.17	20.46	20.19	15.47	26.17	27.69	37.31	25.02	17.9	13.27	15.69	14.5	8.72	7.91	15.88	28.62
**sun [hour]**	73.5	75.5	58	105	146.5	146.5	259	265.5	376	362.5	420.5	282.5	420.5	340.5	329	295	246	232.5	161	216	101.5	135	44	27

**year**	2019																							
**month**	I		II		III		IV		V		VI		VII		VIII		IX		X		XI		XII	
**site**	SE	GER	SE	GER	SE	GER	SE	GER	SE	GER	SE	GER	SE	GER	SE	GER	SE	GER	SE	GER	SE	GER	SE	GER
**max temp [°C]**	-2	3	3	6	5	8	12	12	15	13	22	22	21	20	21	22	15	17	8	13	4	8	3	5
**min temp [°C]**	-7	0	-2	2	-1	4	3	5	6	7	12	14	11	14	13	15	8	12	3	8	-1	4	-3	1
**avg temp [°C]**	-3	2	1	5	3	6	9	10	12	11	20	19	19	18	19	20	13	15	6	11	3	6	2	4
**rain [mm/cm2]**	48.2	96.3	41.7	51.1	70.8	99.5	8.7	40.2	58.2	61.5	49.4	123.2	97.9	105.6	91.6	95.1	43.6	125.9	109.4	115.3	52.3	105.7	79.3	59
**sun [hour]**	108	99	110	121.5	140	125.5	262.5	296.5	241	211	400.5	294.5	317	212.5	258	265.5	194	176.5	95	127	60.5	96	60.5	121

Table 2.

Selective media (NBRIP, PVK, DCP) used for selection of Phoshate solubilizing fungi.

NBRIP		PVK		DCP	
**Ingredient**	**Amount per 1 liter**	**Ingredient**	**Amount per 1 liter**	**Ingredient**	**Amount per 1 liter**
**glucose**	10 g	**glucose**	10 g	**glucose**	10 g
**$Ca_3(PO_4)_2$**	5 g	**$Ca_3(PO_4)_2$**	5 g	**NH_4Cl**	5 g
**$(NH_4)_2SO_4$**	0.5 g	**$MgCl_2$ $6H_2O$**	5 g	**NaCl**	1 g
**NaCl**	0.2 g	**$MgSO_4$ $7H_2O$**	0.25 g	**$MgSO_4$ $7H_2O$**	1g
**$MgSO_4$ $7H_2O$**	0.1 g	**KCl**	0.2 g	**10 % $CaCl_2$***	100 ml
**KCl**	0.2 g	**$(NH_4)_2SO_4$**	0.1 g	**10% K_2HPO_4***	50 ml
**yeastextract**	0.5 g	**pH**	7	**pH**	6,8
**$FeSO_4$ $7H_2O$**	0.002 g				
**pH**	7				

Table 3. Fungal identification table with Accession numbers. S- Sweden, G- Germany, L – Loden, T – Tora, LT – mixture, E – endophyte, R – rhizosphere fungi

Symbol	Indentified as	Country	Genotype	Place of isolation	Accession number
F1	*Penicillium chalabudae*	S	L	E	MW342736
F3	*Penicillium chrysogenum*	S	L	E	MW342737
F4	*Penicillium sp.*	S	L	E	MW342738
F5	*Penicillium chalabudae*	S	T	E	MW342739
F6	*Talaromyces sp.*	S	L	E	MW342740
F7	*Juxtiphoma eupyrena*	S	L	E	MW342741
F12	*Penicillium spinulosum*	G	T	R	MW342742
F13	*Cladosporium sp.*	G	LT	R	MW342743
F14	*Cladosporium pseudocladosporioides*	G	T	R	MW342744
F15	*Penicillium aurantiogriseum*	G	T	R	MW342745
F16	*Clonostachys rosea*	G	T	E	MW342746
F17	*Alternaria sp.*	G	T	E	MW342747
F19	*Gibellulopsis nigrescens*	G	T	R	MW342748
F22	*Alternaria alternata*	G	T	R	MW342749
F23	*Clonostachys rosea*	G	T	R	MW342750
F24	*Clonostachys rosea*	G	T	R	MW342751
F25	*Penicillium bialowiezense*	G	LT	R	MW342752
F27	*Penicillium chrysogenum*	S	L	R	MW342753
F30	*Penicillium restrictum*	S	T	R	MW342754
F32	*Gibellulopsis nigrescens*	G	T	R	MW342755
F33	*Penicillium rubens*	S	L	E	MW342756
F34	*Beauveria bassiana*	S	LT	E	MW342757

Figure 1: Average temperatures and average rainfalls in Rostock (Germany) and Uppsala (Sweden) in 2018 and 2019.

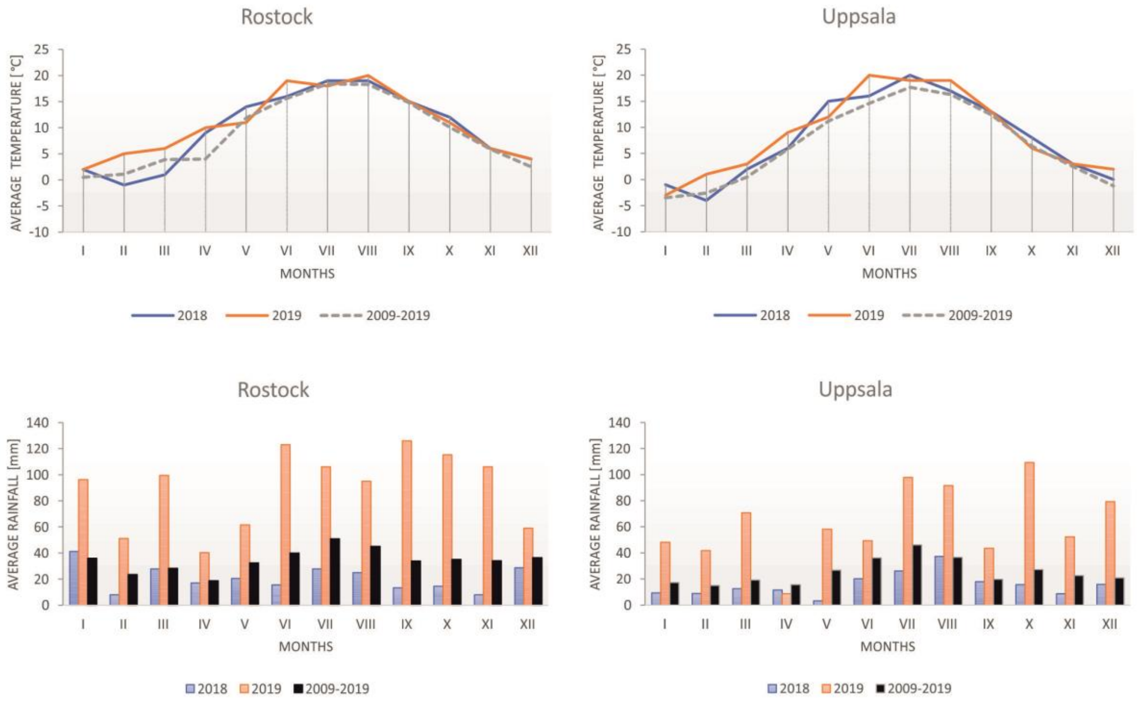

Figure 2: Fungi community structure at the level of Phyla among rhizosphere soil and root samples, two test sites and two seasons.

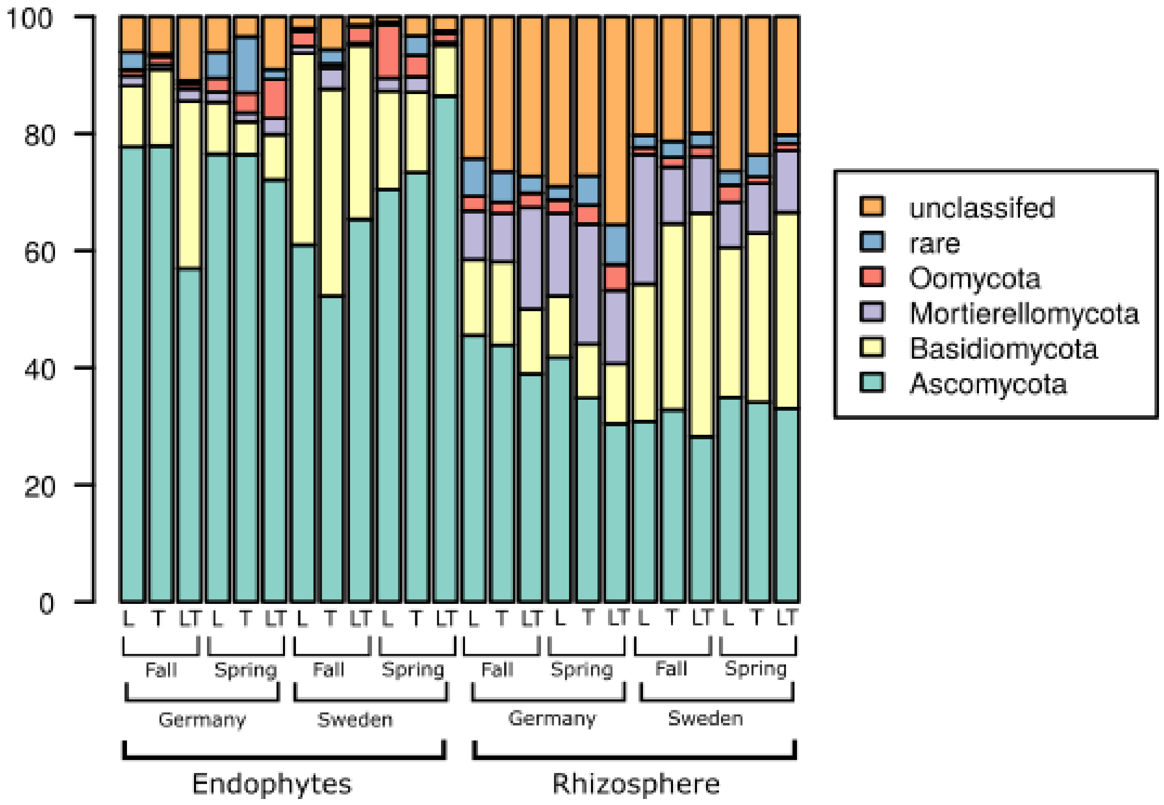

Supplement: Supplementary Figure 1 — Average temperatures and average rainfalls in Rostock (Germany) and Uppsala (Sweden) in 2018 and 2019. [file Data_Sheet_1.PDF]
